# Predictors and risk factors for suicide in late-life depression: a systematic review and meta-analysis

**DOI:** 10.3389/fpsyt.2025.1636838

**Published:** 2025-08-05

**Authors:** Lei Yang, Ping Zhang, Hao Zhang, Jinfeng Wang, Yingzhe Zhou, Donghong Zhang, Yue Zhang, Qing Wen

**Affiliations:** ^1^ School of Nursing, Henan Medical University, Xinxiang, Henan, China; ^2^ Department of Medical Engineering, The Second Affiliated Hospital of Henan Medical University, Xinxiang, Henan, China

**Keywords:** late-life depression, suicide, risk factors, meta-analysis, systematic review

## Abstract

**Background:**

The prevalence of late-life depression (LLD) is high, and its most dangerous, serious, and fatal comorbidity is suicide. Therefore, the present study systematically investigates the risk factors for suicide in individuals with LLD, offering empirical support for the development of preventive interventions against suicidal behavior.

**Methods:**

PubMed, Web of Science, the Cochrane Library, PsycInfo, CNKI, Wan Fang Data, VIP, and CBM databases were searched from the inception of each database to February 2025 to identify observational studies of risk factors for suicide in LLD patients. The Newcastle–Ottawa Scale (NOS) and the Agency for Healthcare Research and Quality (AHRQ) were used to ensure study quality. Stata 18.0 software was used to perform a meta-analysis and sensitivity analysis to compute the pooled odds ratio.

**Results:**

A total of 12 studies (eight case–control, two cross-sectional, and two longitudinal studies), with a quality level of medium or above, were included in the analysis. Depression severity (OR = 3.485, 95% CI: 1.385 to 8.769, *P* = 0.008) was identified as a significant risk factor for suicide in LLD. The age of onset (OR = 0.969, 95% CI: 0.905 to 1.039, *P* = 0.378) was not statistically significant for the risk of suicide in LLD. The descriptive analysis revealed that suicidal ideation, educational level, N3 sleep duration, odor identification dysfunction, alcohol drinking history, cognitive function, history of major trauma, history of suicide attempts, and high-density lipoprotein were associated with an increased suicide risk in LLD.

**Conclusion:**

Our meta-analysis has revealed a variety of factors influencing suicide risk in LLD patients. Clinical staff should strengthen the assessment and screening of risk factors and take timely intervention and targeted treatment to reduce the risk of suicide in LLD.

**Systematic review registration:**

https://www.crd.york.ac.uk/prospero/, identifier CRD420251040029.

## 1 Introduction

The latest data released by China’s National Bureau of Statistics showed that, in 2024, the population aged 60 and above reached 310.31 million, accounting for 22.0% of the total population. Of these, 90.08 million (6.4%) were aged 60–65, and 220.23 million (15.64%) were 65 or older, reflecting an increasingly serious aging population problem. As one of the most common psychiatric disorders in old adults (1), late-life depression (LLD) exhibits a rising prevalence with age (2). Recent studies indicate that its average prevalence has reached 31.74%. With accelerated population aging, the incidence of LLD has increased significantly (3), drawing growing attention from researchers.

Broadly speaking, LLD typically encompasses all individuals with depressive disorders occurring during the older adult stage (≥60) (4). It can be categorized into early-onset depression and late-onset depression depending on the age of first diagnosis (5). LLD poses a significant health risk among the elderly. It is clinically characterized by a persistent depressive mood as the core symptom, accompanied by psychomotor retardation (including slowed thinking and reduced verbal communication), marked anhedonia, somatic complaints, and appetite disturbance. These symptoms occur independently of somatic illnesses or organic brain lesions (6). It is characterized by high rates of disability, high prevalence, long treatment cycles, high costs, increased risk of dementia, high rates of relapse, high rates of disability, high risk of suicide, and high mortality (7).

People with depression are 21 times more likely to commit suicide than the general population (8), and the older the person, the higher the risk (9).Notably, suicide represents the most severe comorbidity of LLD and its most fatal outcome (4), and studies have shown that the factor most associated with suicide attempts in older people is also depression (10). Compared with younger people, LLD patients are also more likely to have recurrent and persistent suicidal ideation, to be more premeditated suicide, and to use more lethal methods of suicide (11). Relevant data indicate that LLD patients have a significantly higher suicide risk compared with an age-matched healthy population (4). Approximately 60% of LLD patients exhibit suicide risk, which is defined as the probability of engaging in suicidal behavior based on the presence of suicidal ideation. This metric serves as a key predictor in suicide risk assessment and clinical intervention (12). LLD patients with suicidal behavior seriously jeopardize the lives and health of patients, and it is also related to the poor prognosis of the patients. This causes devastating psychological trauma to families and increases healthcare costs.

In recent years, there has been a significant growth in research investigating the risk factors for suicide in LLD patients. However, the findings of different studies are discrepant (13). To improve strategic intervention and prognosis, a systemic analysis of factors contributing to suicide risk and the clear identification of LLD patients with suicide risk are indispensable. Current research had primarily focused on meta-analyses of depression across all age groups (14, 15), while existing reviews on suicide risk factors in patients with LLD had been largely limited to qualitative descriptions (16), lacking systematic quantitative investigation through meta-analysis of suicide risk factors specifically in LLD populations. Thus, this meta-analysis was conducted to estimate risk factors and provide more reliable evidence for the clinical setting.

## 2 Methods

This systematic review and meta-analysis followed the Preferred Reporting Items for Systematic Reviews and Meta-analyses (PRISMA) 2020 reporting guideline (17) and was registered in the PROSPERO platform under the registration number CRD420251040029.

### 2.1 Search strategy

We conducted literature searches across PubMed, Web of Science, the Cochrane Library, PsycInfo, CNKI, Wan Fang Data, VIP, and CBM databases from the inception of each database until February 20, 2025. The retrieval approach of the combination of free words and subject words was adopted and with the search strategy adjusted according to the search rules of each database. Terms related to the words “depressive disorders”, depression, “depressive disorder”, geriatric, senile, elderly, “late-life”, suicide risk, suicide-related behavior, suicide ideation, “suicide attempts”, impact factor, contributing factor, and risk factor were used to retrieve potentially relevant articles on the risk factors for suicide in LLD patients. The literature retrieval strategy takes PubMed as examples. The comprehensive PubMed search strategy is delineated in **Table 1**. The detailed search strategy is provided in the **Supplementary Material**.

**Table 1:** Search strategy for PubMed.

#1	“Depressive Disorder”[Mesh] OR depression [Title] OR “depressive disorders”[Title] OR “depressive disorder”[Title] OR “disorders, depressive”[Title] OR “disorder, depressive”[Title]
#2	“Aged”[Mesh] OR Aging [Title] OR elderly [Title] OR geriatric [Title] OR senile [Title] OR older [Title] OR “old age”[Title] OR “older adult*”[Title] OR “late-life”[Title] OR “late in life”[Title] OR “late-onset”[Title]
#3	“Suicide”[Mesh]) OR suicide risk [Title] OR suicide related behavior [Title] OR suicide ideation [Title] OR “suicide thought”[Title] OR “suicidal thought”[Title] OR “suicidal ideation”[Title] OR “suicide behavior”[Title] OR “‘suicide attempts”[Title] OR “attempted suicide”[Title] OR suicidal attempts [Title] OR suicide [Title]
#4	“Risk Factors”[Mesh] OR impact factor [Title] OR contributing factor [Title] OR related factor [Title] OR relevant factor [Title] OR influencing factor [Title] OR risk factor [Title] OR associat*[Title] OR correlat*[Title] OR determin*[Title] OR predict*[Title] OR interact*[Title] OR mediat*[Title]
#5	#1 AND #2 AND #3 AND #4

### 2.2 Selection criteria

Inclusion criteria: (1) The study subjects were patients with depression as diagnosed by the Second Revision of the Chinese Classification Scheme and Diagnostic Criteria for Mental Diseases, the Diagnostic and Statistical Manual of Mental Disorders, 4th edition (DSM-IV), the Diagnostic and Statistical Manual of Mental Disorders, 5th edition (DSM-V), or the International Classification of Diseases-10 (ICD-10); (2) Age ≥60 years; (3) Type of study was case–control study, cohort study, and cross-sectional study; and (4) The study was on the risk factors for suicide in LLD patients, with a clear or calculable odds ratio (OR) and 95% confidence intervals (CIs) of each influencing factor of suicide risk.

Exclusion criteria: (1) review, case reports, or conference papers and other types of research, (2) non-Chinese and English literature, (3) literature with no relevant data, incomplete data that cannot be extracted, and (4) duplicate publication, the literature cannot be obtained in full text.

### 2.3 Study screening and data extraction

Literature search, checking, and screening were completed by two researchers independently using NoteExpress software, extracting data from the included literature and cross-checking them, and in cases of doubt or disagreement, the study was evaluated by a third reviewer until an interactive consensus was reached on the inclusion criteria. The first author, year of publication, where the study was conducted, diagnostic criteria for late-life depression, study design, sample size, influencing factors, definition (at least three relevant articles for each risk factor), odds ratio (OR), and 95% confidence interval (95% CI) were abstracted from the included studies to Microsoft Excel 2016. If the studies did not report OR equivalent measures, raw data were screened to determine whether ORs could be calculated.

### 2.4 Assessment of suicide risk and associated factors

The severity of depression was rated using the Hamilton Rating Scale for Depression (HAMD-17). Age of onset was defined as the age at first occurrence of depression meeting the diagnostic criteria. Suicidal behavior refers to the act of intentionally ending one’s own life, encompassing the processes of suicidal ideation, suicide planning, suicide attempt, and completed suicide. Suicide risk refers to the likelihood of an individual exhibiting a suicidal behavior based on the presence of suicidal ideation (18). Patients were at risk for suicide when they presented with any one of suicidal ideation, suicidal planning, or suicidal behavior (19).

### 2.5 Quality assessment

Two systematically trained researchers evaluated the methodological quality of the included literature and cross-checked the results, asking a third researcher to assist in the judgment if disagreements were encountered. For cohort and case–control studies, the Newcastle–Ottawa Scale (NOS) (20) was used. The quality of the study was evaluated by eight items under the three categories of participant selection, comparability of study groups, and ascertainment of outcome or exposure. A score of ≥7 is classified as high-quality literature, 5 to 6 is classified as moderate-quality literature, and ≤4 is classified as low-quality literature. For the quality assessment of a cross-sectional study, the 11-item criteria recommended by the US Agency for Healthcare Quality and Research (AHRQ) were used (21). A score of 0 to 3 indicates low quality, 4 to 7 indicates medium quality, and 8 to 11 indicates high quality.

### 2.6 Statistical analysis

Statistical analysis was performed using Stata 18.0 software. Data expressed in the form of means were converted to ORs using the method recommended by the Cochrane Handbook of Systematic Reviews. Results were reported as OR and 95% CI. The *χ* ^2^ test was used to evaluate the heterogeneity of the included studies (the test level was *α* = 0.1), and the size of the heterogeneity was assessed in combination with *I* ^2^. When *I* ^2^ <50% and *P >*0.05, it showed that there was less heterogeneity among the studies, the fixed-effects model would be used to combine the effect sizes. If *I* ^2^ ≥50%, *P* < 0.05, it indicated high heterogeneity between studies. Effect sizes were combined using a random-effects model. When heterogeneity was large, sensitivity analyses were further conducted by comparing the consistency of the results of the random and fixed-effects models as well as by the leave-one-out analysis. Descriptive analysis was adopted for the influencing factors that were not suitable for meta-analysis. When three or more papers were included, publication bias was performed by quantitative analyses based on Begg’s and Egger’s test. *P >*0.05 indicated that there was no publication bias. *P <*0.05 was considered statistically significant.

## 3 Results

### 3.1 Study selection

**Figure 1:**
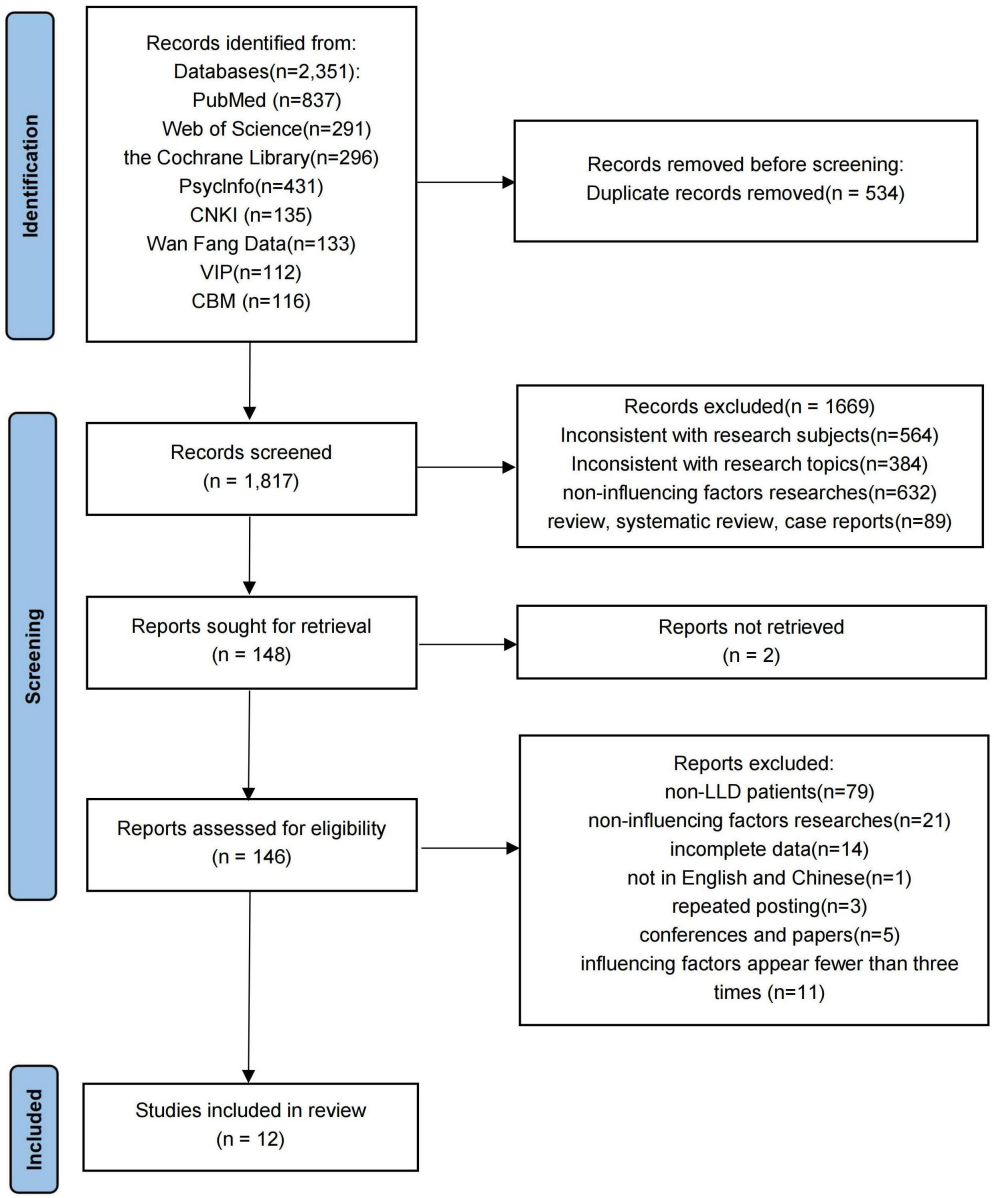
PRISMA (2020) flow chart of the study selection process.

From the total of 2,351 relevant articles retrieved, 534 duplicate literatures were eliminated, and 1,669 articles were removed after reviewing the titles and abstracts. These studies were excluded because their research subjects and designs did not meet the inclusion criteria and because they did not focus on suicide and suicidal-related factors. Then, the remaining 148 literatures were evaluated by reviewing the full text, and 12 literatures (13, 22–32) had finally remained. **Figure 1** outlines the systematic literature screening process.

### 3.2 Characteristics of the included studies and quality evaluation

Of the 12 studies eventually included, eight were case–control studies, two were cross-sectional studies, and two were longitudinal studies. Seven studies were conducted in China, three studies were conducted in America, and one study each was conducted in Canada and Greece. The total cumulative sample size was 2,324 cases. The results of literature quality evaluation showed that eight studies (13, 22, 23, 25–27, 29, 30) were of high quality and four studies (24, 28, 31, 32) were of moderate quality. The basic characteristics of all 12 included literatures are illustrated in **Table 2**.

**Table 2:** Characteristics and quality evaluation of the included studies.

Author	Study period	Country	Diagnostic Criteria	Study design	Sample size (case/control)	Factors	Quality evaluation
Zhu et al.	2023	China	C	Case–control study	161/82	1, 2, 3	High
Chen et al.	2023	China	A	Case–control study	81/47	1, 4, 5, 6, 7, 8	High
Su et al.	1998	China	D	Cross-sectional study	36/107	1, 9, 10	Medium
Qin et al.	2023	China	C	Case–control study	110/90	1, 11, 12, 13, 14	High
Zhu et al.	2024	China	C	Case–control study	66/37	15, 16	High
Olgiati et al.	2024	US	A	Case–control study	23/235	15, 17, 18, 19, 20	High
Liu et al.	2023	China	B	Case–control study	48/35	15, 21, 22	Medium
Xu et al.	2024	China	A	Case–control study	63/262	1, 23, 24, 25, 26, 27, 28, 29	High
Stephane et al.	2021	Canada	A	Case–control study	43/29	1, 30, 31, 32	High
Szanto et al.	2020	US	A	Longitudinal study	311	1, 15, 22, 33, 34, 35, 36	Medium
Rossetos et al.	2019	Greece	A	Cross-sectional study	17/48	15, 37	High
Natalie et al.	2014	US	A	Longitudinal study	23/223	15, 21, 38, 39, 40	Medium

Diagnostic criteria: A, DSM-IV; B, DSM-V; C, ICD-10; D, The Second Revision of the Chinese Classification Scheme and Diagnostic Criteria for Mental Diseases. Factors: 1, HAMD-17; 2, history of alcohol consumption; 3, history of major trauma; 4, sleep efficiency; 5, total sleep duration; 6, number of awakening; 7, non-rapid eye movement stage 1 sleep duration; 8, non-rapid eye movement stage 3 (N3) sleep duration; 9, spirit; 10, family history; 11, loneliness or feeling of worthlessness; 12, family issues; 13, anxiety; 14, Self-Rating Idea of Suicide Scale (SIOSS); 15, age of onset; 16, vocabulary learning; 17, verbal fluency; 18, attention index; 19, HDL; 20, dyskinesia; 21, number of lifetime episodes; 22, Beck Scale for Suicide Ideation (BSI); 23, odor identification dysfunction; 24, global cognition (MMSE); 25, information processing speed; 26, memory; 27, language; 28, executive function; 29, visuospatial ability; 30, MoCA total score; 31, delayed recall subtest; 32, trail making subtest; 33, gender; 34, introversion; 35, history of suicide attempt; 36, deficits in cognitive control; 37, guilt delusions; 38, education; 39, number of psychotic symptoms; 40, current suicidal ideation (DIS). DIS, National Institute of Mental Health Diagnostic Interview Schedule.

### 3.3 Meta-analysis and descriptive analysis

#### 3.3.1 Meta-analysis

**Figure 2:**
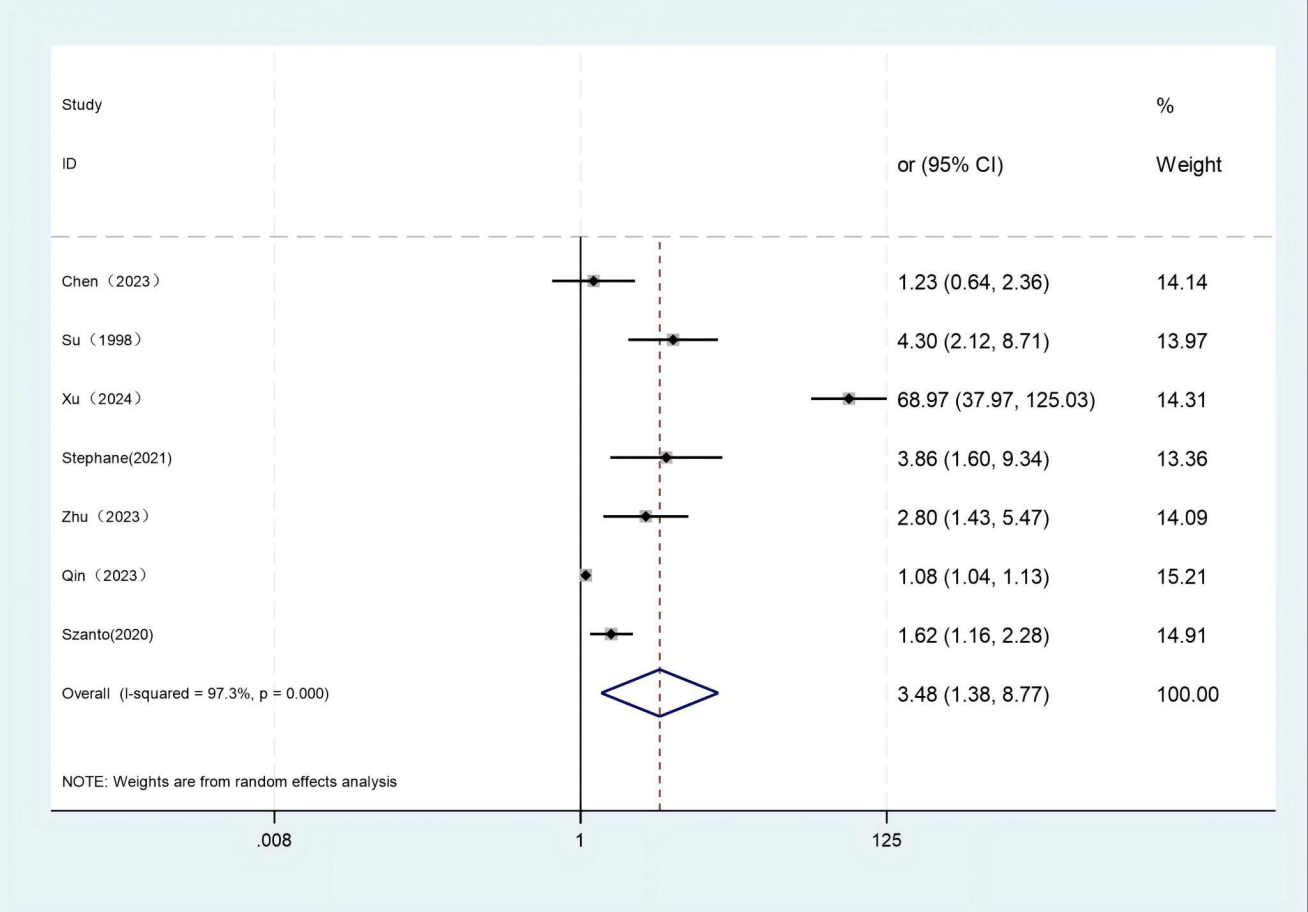
Forest plot of depression severity. Test for heterogeneity *I* ^2^ = 97.3%, *P* = 0.000. The random-effect model was used. The overall effect *P* = 0.008 < 0.05 shows that depression severity is a risk factor for suicide in late-life depression.

**Figure 3:**
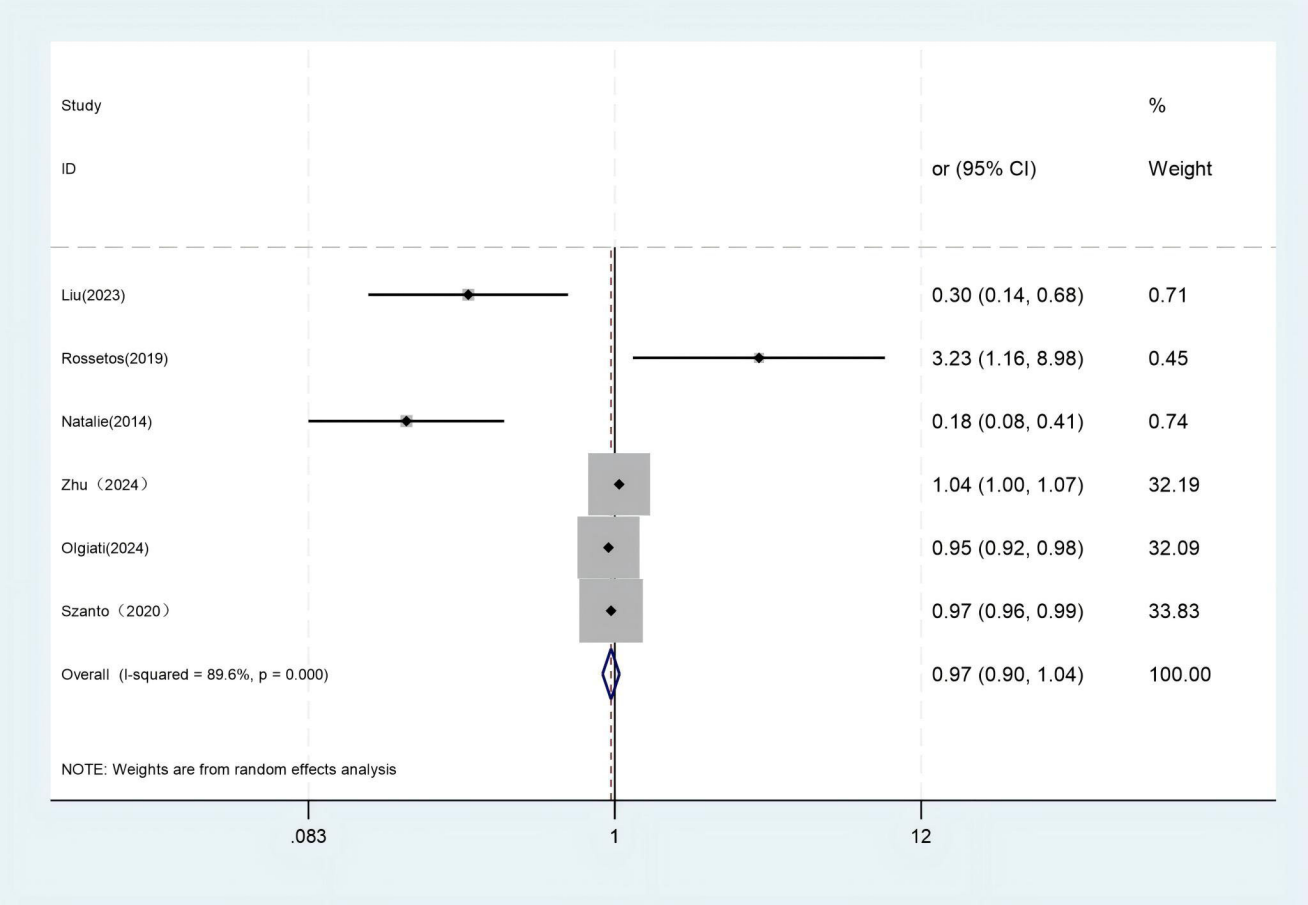
Forest plot of age of onset. Test for heterogeneity *I* ^2^ = 89.6%, *P* = 0.000. The random-effect model was used. The overall effect *P* = 0.378 > 0.05 shows that age of onset is not an influencing factor of suicide risk in late-life depression.

A total of 40 factors related to the risk of suicide in LLD patients were addressed in the literature included in this study, and after exclusion screening, two influencing factors were entered into a meta-analysis. Due to significant heterogeneity across studies in depression severity and age at onset, a random-effects meta-analytic approach was employed. The results showed that depression severity (OR = 3.485, 95% CI: 1.385 to 8.769) was a risk factor for suicide in LLD (*P* < 0.05). The age of onset (OR = 0.969, 95% CI: 0.905 to 1.039, *P* = 0.378) was not statistically significant for the risk of suicide in LLD (*P* = 0.378). The pooled estimates and heterogeneity statistics from the meta-analysis are detailed in **Table 3**. Forest plots depicting the meta-analysis of depression severity and age of onset effects on suicide risk in LLD patients are shown in **Figures 2** and **3**.

**Table 3:** Meta-analysis of the risk factors for suicide in late-life depression.

Factors	Number of studies	Heterogeneity		Analytical model	Summary effect estimate		
		*I* ^2^ (%)	*P*-value		OR	95% CI	*P*-value
Depression severity (severe)	7	97.3	<0.001	RE	3.485	1.385–8.769	0.008
Age of onset (young)	6	89.6	<0.001	RE	0.969	0.905–1.039	0.378

RE, random-effects model; FE, fixed-effects model.

#### 3.3.2 Descriptive analysis

Five articles (26, 27, 29–31) in the included literature reported the relationship between cognitive functioning and suicide risk, with memory, visuospatial ability, verbal fluency, vocabulary learning, executive functioning, and information processing speed being negatively correlated with the risk of suicide in LLD patients. Three articles reported that suicidal ideation (25, 28, 31) was associated with suicide risk. Two articles reported that the number of lifetime episodes (28, 32) was associated with suicide risk. A single study pointed out that sleep inefficiency, decreased total sleep time and increased number of awakenings (23), reduced non-rapid eye movement stage 3 (N3) sleep duration (23), feelings of loneliness or worthlessness, severe family issues and high levels of anxiety (25), high-density lipoproteins and more dyskinesias (27), educational levels (32), and odor identification dysfunction (29) are risk factors for suicide in LLD patients. History of alcohol consumption and major trauma (22), family history (24), history of suicide attempt (31), and guilt delusions (13) are risk factors for suicide in LLD patients.

### 3.4 Sensitivity analysis

#### 3.4.1 Changing effects model

The sensitivity analyses of the combined ORs included the influencing factors, and their 95% CIs were derived using a different-effects model. The results showed good consistency in depression severity, indicating stable findings. Conversely, the age of onset exhibited an inverse pattern. The results of the change-effects model are shown in **Table 4**.

**Table 4:** Sensitivity analysis of two models to the influencing factors of suicide risk.

Factors	Fixed-effects model		Random-effects model		Stability
	OR	95% CI	OR	95% CI	
Depression severity (severe)	1.122	1.079–1.167	3.485	1.385–8.769	Stability
Age of onset (young)	0.977	0.965–0.989	0.969	0.905–1.039	Instability

#### 3.4.2 Leave-one-out analysis

Sensitivity analyses of studies with *I* ^2^ >50% and more than two articles in the influencing factors were conducted by excluding individual studies through the one-by-one exclusion method. It was found that the heterogeneity of depression severity and age of onset were reduced through the exclusion of the literature but did not change significantly, and there was no directional change in the results of the analyses, which suggests that the findings are basically reliable. The results of the sensitivity analysis are shown in **Table 5**.

**Table 5:** Exclusion analysis of the influencing factors of suicide risk.

Factors	Excluding literature	Pre-exclusion				Post-exclusion			
		*I* ^2^ (%)	Model	OR (95% CI)	*P*-value	*I* ^2^ (%)	Model	OR (95% CI)	*P*-value
Depression severity (severe)	1	97.3	RE	3.485 (1.385–8.769)	0.008	85.8	RE	1.976 (1.253–3.119)	0.003
Age of onset (young)	2	89.6	RE	0.969 (0.905–1.039)	0.378	88.4	RE	0.931 (0.856–1.014)	0.099

RE, random-effects model.

### 3.5 Publication bias

**Figure 4:**
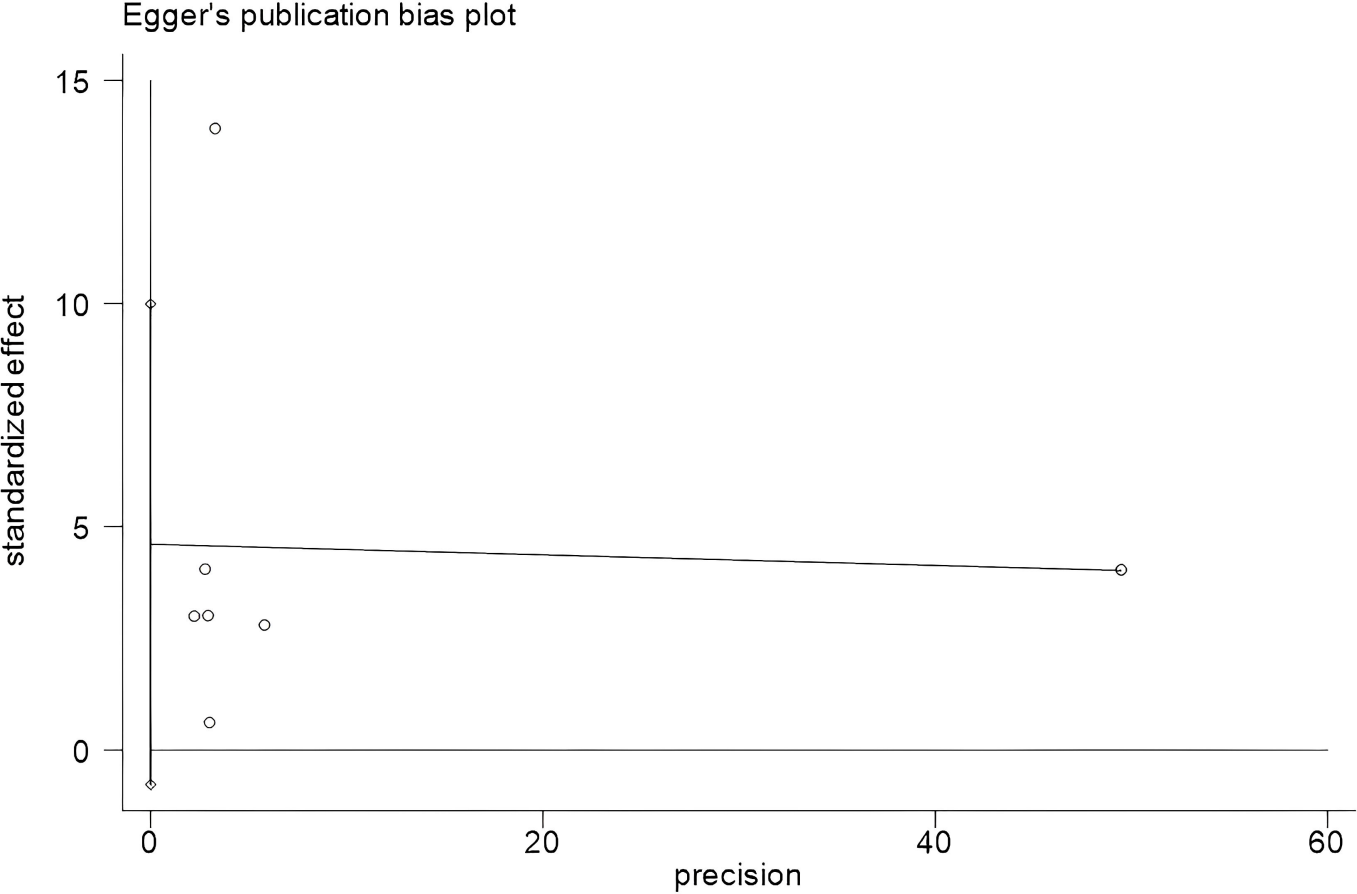
Egger’s plot of depression severity. Begg’s test showed Pr > |z| = 0.230; Egger’s test showed *P* = 0.079, *P* > 0.05. The result showed that there was no significant publication bias.

**Figure 5:**
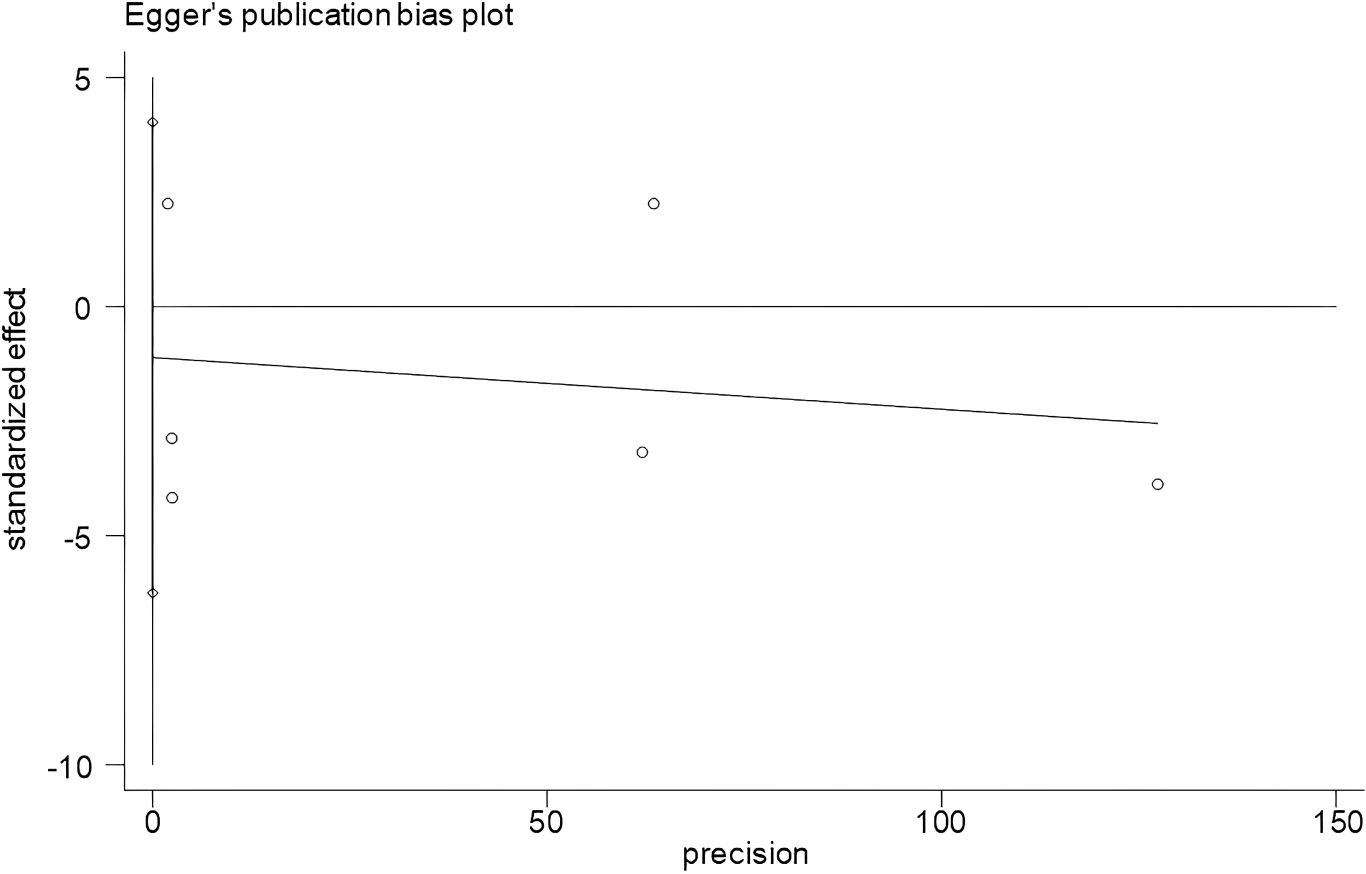
Egger’s plot of age of onset. Begg’s test showed Pr > |z| = 0.707; Egger’s test showed *P* = 0.579, *P* > 0.05. The result showed that there was no significant publication bias.

Publication bias analysis of the influential factors using Begg’s and Egger’s test in Stata 18.0 software showed that no significant bias was seen in depression severity (*t* = 2.20, *P* = 0.079) and age of onset (*t* = -0.60, *P* = 0.579), as shown in **Table 6** and **Figures 4** and **5**.

**Table 6:** Egger’s test for influencing factors.

Factors	*T*	*P*
Depression severity	2.20	0.079
Age of onset	-0.60	0.579

## 4 Discussion

### 4.1 Relationship between depression and suicide risk

Our study showed that depression severity is a risk factor for suicide in LLD patients. The greater the degree of depression in LLD patients, the greater the suicidal ideation in LLD patients due to the aggravation of pain, despair, negative emotions brought about by depression, and the impairment of cognitive functioning. Depression severity has been consistently identified as the most robust predictor of suicidal ideation in older adult populations across multiple studies (33). Moreover, the more severe the degree of depression and the more severe the clinical symptoms, the more prolonged and difficult it is to cure the disease, which will significantly increase the patient’s sense of despair and thus increase the patient’s risk of suicide. It has also been shown that LLD patients who have attempted suicide are more depressed (32). This is consistent with previous findings that depressive severity is strongly associated with a higher suicidal behavior (34, 35). The medical staff should focus on the people with higher levels of depression, pay attention to the changes in the level of depression, establish a trusting relationship with the patients, and assist them in actively cooperating with the treatment to reduce the level of depression and reduce the risk of suicide.

### 4.2 Relationship between age of onset and suicide risk

Previous studies have shown that the age of onset of depression in elderly patients with suicide attempts is younger than in elderly patients with depression without suicide attempt (32). Compared with late-onset depression patients, elderly individuals with early-onset depression who experienced their first episode before the age of 60 exhibit a higher recurrence rate, a greater number of prior episodes, and a higher prevalence of suicide attempt history (36). However, it has also been noted that patients with suicide attempt have a greater age of onset and show a positive correlation between age of onset and depression severity scores (13). In our study, the age of onset was not significantly associated with suicide risk in LLD patients. This may be due to the greater heterogeneity of the included studies. Current evidence remains inconclusive regarding this association. Future high-quality studies are still needed to elucidate the impact of age at first onset on suicide risk in late-life depression.

### 4.3 Relationship between relevant influences and suicide risk in descriptive analysis

#### 4.3.1 Educational levels

Previous studies have shown that LLD patients with a low level of education have limited cognitive reserves and a relatively poor ability to learn and master new knowledge independently, and when faced with physiological changes and psychological stress triggered by diseases, they lack a systematic foundation of medical knowledge and effective ways to cope with them, which reduces the effectiveness of treatment and affects the regression and recovery of the disease, which greatly increases the physical and mental burden of the patients and seriously reduces their quality of life, leading to an increased risk of suicide. Lower education is associated with an increased suicide risk of LLD relative to higher education (37), which was consistent with the results of previous studies (16, 38). Studies have shown that educational attainment is negatively correlated with depression among the elderly (39). The relevant meta-analysis also proved that low educational level is a suicide risk factor for LLD (40). Lower educational levels can exacerbate the depressive symptoms in this population, thus increasing their suicide risk. Due to the small amount of literature included in this study on educational attainment, more large-sample studies are needed in the future to validate the results and obtain stronger conclusions.

#### 4.3.2 Suicidal ideation

The intensity of suicidal ideation is a risk factor for suicide risk in LLD patients. With the continuous enrichment of suicide theories, studies have found that suicidal behavior is not a simple act but a continuous and complex process from suicidal ideation to suicidal death (41). Suicide ideation is a crucial stepping stone on the path to suicide completion (42). Relevant studies have shown that if a patient has suicidal ideation, it leads to a rapid increase in the risk of suicide. Specifically, each unit increase in suicidal ideation severity is associated with a 4.408-fold higher odds of suicide attempt (43). Moreover, some data show that about 60% of depressed patients who first experience suicidal ideation develop a planned suicidal behavior within 1 year (44). It has also been shown that LLD patients with suicidal ideation have more severe depressive symptoms (45, 46) and higher relapse rates than those without suicidal ideation (47). The high rates of relapse and severe depressive symptoms increase the risk of suicide in LLD patients. Current research indicates that older adults may struggle to communicate effectively with healthcare providers due to disease-related impairments and may conceal suicidal ideation because of the sensitive nature of the topic. Therefore, clinicians should closely monitor the patients’ emotional states and integrate known risk factors for suicidal ideation to promptly identify at-risk individuals and implement targeted interventions (29, 30). This approach can mitigate the impact of suicidal ideation on disease progression while preventing its escalation into suicidal behaviors or completed suicide.

#### 4.3.3 Number of lifetime episodes

The number of lifetime episodes is a risk factor for suicide in LLD patients. Repeated episodes of the disease seriously affect the patients’ quality of life, frustrate their confidence in fighting the disease, and increase the burden on families and society. Depression is expected to be one of the diseases with the highest burden of disease in China by 2030 (48). Studies have shown that the burden of care for depressed patients in China presents a moderate level (49). Depressed patients have a long treatment cycle and require long-term medication and regular follow-ups, and their caregivers bear a greater financial burden and other caregiving burdens. A greater number of lifetime episodes creates a greater caregiving burden, which has a huge impact on the physical and mental health of caregivers (50). Depression makes patients more susceptible to adverse life events (51), causing individuals to pay more attention to negative information and thoughts and to negatively evaluate and process the stresses and dilemmas the face, which enhance and maintain the negative emotions associated with perceived stress, and ultimately the individual is overwhelmed with suicidal thoughts (16). Persistent or recurrent moderate to severe depressive episodes in LLD patients can seriously worsen the patient’s condition, escalating to suicidal tendency in the most severe cases (4).

#### 4.3.4 N3 sleep duration

Previous studies have shown that shortening the N3 sleep duration is a risk factor for suicide in LLD patients. Adequate N3 sleep duration facilitates energy restoration and mental health maintenance. Notably, in individuals with psychological disorders, healthy sleep patterns may enable temporary psychological disengagement from waking-hour stressors. Individuals may be more susceptible to depressive moods during nighttime hours due to the reduced availability of social support systems, which can exacerbate their experience of psychological distress, which, in turn, can lead to exacerbation of the condition. This phenomenon suggests a complex interaction between sleep and depression (52). Studies have shown that patients with major depression are, in turn, associated with an increased risk of suicidal and non-suicidal self-harm (53).

#### 4.3.5 Other influencing factors

It has been suggested that co-morbid anxiety and depression may pose a greater suicide risk than depression alone (42), possibly because the presence of anxiety symptoms in LLD patients predicts more severe depressive and somatic symptoms, as well as poorer social functioning, and possibly because of the mental anguish associated with the anxiety symptoms and thus an increased risk of suicide in patients (54). Previous studies have shown that sleep disorders were associated with suicide in older adults (55) and were risk factors for suicide in LLD patients. This may be due to the fact that sleep disorders tend to trigger disruptions in an individual’s ability to regulate their emotions, while increased wakefulness time allows negative emotions to persist for a longer period of time, leading to an increased risk of suicide (56). LLD patients with suicidal ideation performed worse on the MoCA total score, especially on the delayed recall test, compared with those without suicidal ideation, possibly due to the fact that cognitive changes may affect attentional biases and problem-solving abilities in LLD patients, putting them at a higher risk for suicidal behavior (30). Studies have also noted that suicide attempts have been recognized as a significant risk factor for suicide completion (10). A history of alcohol consumption is a risk factor for suicide in LLD patients, and studies have shown that the association between alcohol and suicidal behavior is high across all age groups, and this correlation increases significantly, especially in the elderly (57). The risks and problems of alcohol consumption in older adults may exacerbate existing depression, and the frequency and amount of alcohol consumption are inversely associated with depression treatment outcomes. A previous research has also shown that the co-occurrence of alcohol abuse and mood disorders causes a greater risk of suicide (58).

### 4.4 Limitations

As all of the included studies were observational, the results of the meta-analysis of the influencing factors should be kept in research. The heterogeneous operationalization of suicide risk across studies introduces measurement bias that may limit the generalizability of our findings. The limited sample size and heterogeneity in measurement methodologies may have constrained the statistical power of our findings, and more high-quality studies and more sample sizes are still needed to further validate and supplement the results in the future. Concurrently, this study has not adequately parsed the potential moderating effects of cultural and regional disparities. Given that contextual factors such as cross-cultural attitudes toward suicide and regional variations in social support systems were not fully incorporated as covariates, interpretive biases may arise in the aggregated conclusions. Then, the subjects of this study were not differentiated into early-onset and late-onset types. In the future, a classification-based discussion can be conducted to explore the influencing factors of suicide risk in patients with early-onset depression and late-onset depression to implement targeted interventions.

## 5 Conclusion

Depression severity is identified as a significant risk factor of suicide in LLD patients. The age of onset is not statistically significant for suicide risk. In addition, a few influences not included in the meta-analysis, such as N3 sleep duration, number of lifetime episodes, suicidal ideation, educational levels, cognitive functioning, high levels of anxiety, odor recognition dysfunction, history of alcohol consumption, history of major trauma, and history of suicide attempts, are also risk factors for suicide risk in LLD patients. Therefore, clinical staff should strengthen the assessment and screening of these factors, and we can construct a predictive model based on the influencing factors of suicidal risk. The model can predict the probability of patients developing a suicidal risk, thereby helping to identify high-risk groups and implement targeted intervention measures and ultimately promoting patient prognosis and reducing the medical burden on caregivers and the society.

## Data Availability

The original contributions presented in the study are included in the article/**Supplementary Material**. Further inquiries can be directed to the corresponding author.
